# Image denoising via a non-local patch graph total variation

**DOI:** 10.1371/journal.pone.0226067

**Published:** 2019-12-12

**Authors:** Yan Zhang, Jiasong Wu, Youyong Kong, Gouenou Coatrieux, Huazhong Shu

**Affiliations:** 1 LIST, the Key Laboratory of Computer Network and Information Integration, Southeast University, Ministry of Education, Nanjing, China; 2 Centre de Recherche en Information Biomédicale Sino-Français, Nanjing, China; 3 International Joint Research Laboratory of Information Display and Visualization, Southeast University, Ministry of Education, Nanjing, China; 4 IMT Atlantique, Inserm, LaTIM UMR, Brest, France; University of North Carolina at Chapel Hill, UNITED STATES

## Abstract

Total variation (TV) based models are very popular in image denoising but suffer from some drawbacks. For example, local TV methods often cannot preserve edges and textures well when they face excessive smoothing. Non-local TV methods constitute an alternative, but their computational cost is huge. To overcome these issues, we propose an image denoising method named non-local patch graph total variation (NPGTV). Its main originality stands for the graph total variation method, which combines the total variation with graph signal processing. Schematically, we first construct a *K*-nearest graph from the original image using a non-local patch-based method. Then the model is solved with the Douglas-Rachford Splitting algorithm. By doing so, the image details can be well preserved while being denoised. Experiments conducted on several standard natural images illustrate the effectiveness of our method when compared to some other state-of-the-art denoising methods like classical total variation, non-local means filter (NLM), non-local graph based transform (NLGBT), adaptive graph-based total variation (AGTV).

## Introduction

Image denoising is one of the most fundamental and widely studied problems in low-level image processing. Its main purpose is to reduce undesirable distortions and noise present in images while preserving important features such as discontinuities, edges, corners and textures. Image denoising can be described by the following model [1]:

$$u_{0}=u+z,$$ (1)

where *u* is the original image, *u*_0_ is the observed image and *z* is assumed to be an additive white Gaussian noise.

Image denoising is an ill-posed problem and requires an appropriate regularization to restore the original image [2]. Solutions proposed so far can be divided into two categories [3]. The first one makes use of some prior knowledge about images such as image smoothness or sparsity of image coefficients in certain transformed domains (e.g., DFT or Wavelet). The second one relies on existing self-similarity in images as, for example, bilateral filter and non-local means (*NLM*) filter [4]. The solution we propose in this paper belongs to the first class of method where the denoising problem is expressed as:

$$\underset{x}{\text{min}}\;{\left\|u_{0}-u\right\|}_{2}^{2}+\mu \;\text{prior}(u),$$ (2)

where ${\left\|u_{0}-u\right\|}_{2}^{2}$ is a fidelity term which requires that the desired image *u* approaches to the observation image *u*_0_ while the prior(*u*) is a term representing the prior knowledge we have on the original image *u* and *μ* is a weighting parameter which determines the tradeoff between prior knowledge and the fidelity of the observation.

Total variation (TV) algorithm [5] and its improved versions [6] are among the most popular prior knowledge-based methods due to their efficiency for image denoising. They also constitute powerful tools for multiscale image analysis [7]. Most total variation-based image denoising methods consider the original image as a continuous function defined on ℜ^2^, and evaluate the noise through the integration of the absolute gradient of the observed function. Under this hypothesis, it is then natural to take the image as a smooth function over a discrete sampling structure [8]. Reducing the total variation indicates that the unwanted details have been removed. However, reducing the gradient may not be sufficient in the case of images with noise or with many details.

Recently, image denoising has also been studied from the point of view of graph signal processing (GSP) [9]. Kheradmand and Milanfar [3] proposed a general graph-based regularization framework which was later modified by Pang *et al*. [10] who used patch gradients instead of patch intensities to define the patch self-similarity. Mahmood *et al*. [11] proposed an adaptive graph total variation (AGTV) for tomographic reconstruction. Though these methods provide good experimental performance, some problems remain: (i) there is neither theoretical justification nor intuitive interpretation of the relationship between the graph structure derived from the image and the image denoising performance; (ii) these algorithms involve complicated mathematical construction and large calculations.

In addition, one of the key issues in performing graph signal processing (i.e. denoising in our case) concerns the selection of edge weights [12]. Indeed, these weights have a significant effect on the amount of noise removal. In [13], Smolka *et al*. compute the weights using a Gibbs distribution of the intensities for the adjacent pixels. Black *et al*. [14] derived these weights via robust statistics. A more common but less robust approach exploits a Gaussian kernel function where the weights are calculated only from two isolated pixels based on their intensities and location information [15]. A more reliable idea is to consider the pixel neighborhood due to the high degree of redundancy in natural images [6]. In such a way Buades *et al*. [4] proposed to use a windowed non-local means filter to characterize one pixel instead of only using the pixel itself. Some graph-signal based image denoising methods also borrow the image patch thought to construct the graph, the most typical scheme being AGTV. However, they only take the image patch intensity into consideration and ignore the location information of the patch. Thus, image spatial information has not been utilized.

To overcome the above problems, we propose a non-local patch graph total variation (NPGTV) as a novel method for natural image denoising. Our method can be seen as an improved version of AGTV by considering the pixel coordinate as an ingredient to construct the *K* nearest neighbor graph (KNN graph). More clearly, both the image patch intensity and patch location information are taken into account. By doing so, image details can be preserved at a greatest extent. In addition, in this paper, we also analyze the impact of the patch size and of the *K* value of the KNN graph on the denoising performance. It is important to notice that AGTV reconstructs and utilizes graph total variation, repeatedly, our method merely constructs a non-local patch graph and use GTV model once. In this way, time cost is significantly reduced. As we will see in the sequel, our proposed method can achieve better performance compared to some recent and efficient non-local based denoising methods and total variation based denoising methods.

The rest of this paper is organized as follows. Some basic knowledge about graph signal processing is reviewed in Section 2. Section 3 presents our NPGTV method and details its implementation. In Section 4, we experimentally illustrate its effectiveness and compare it to several state-of-the-art denoising methods. Some future works are sketched in Section 5.

## Prior work

### Graph signal and weighted graph

A dataset consisting of *N* elements with a known relationship between these elements can be represented by a graph *G* = {*V*, *A*, *W*}, where *V*, *A* and *W* stand for the nodes set, the adjacency matrix and the weight matrix of graph *G*, respectively. Each element in set *V* corresponds to a node in graph *G* while each *W*_*i*,*j*_ in *W* reflects the degree of relationship in-between the two nodes *v*_*i*_ and *v*_*j*_. In the general case, *G* can have directed or undirected edges (i.e. arcs) and *W*_*i*,*j*_ can take arbitrary real or complex values. For a denoising image, the direction of edge is meaningless and, considering one node *v*_*i*_ and nodes connected to it, one can construct the neighborhood of *v*_*i*_ as *N*_*i*_ = {*j*|*W*_*i*,*j*_ ≠ 0}.

The graph signal of *G* is defined as a map from the nodes of *G* into the real number set ℜ,

$$\begin{array}{l} V\;\to ℜ\\ v_{i}\to \;f\left(v_{i}\right), \end{array}$$ (3)

where *f* is a real value function and *f*(*v*_*i*_) is the graph signal on vertex *v*_*i*_. A graph signal can also be represented as a vector **f** ∈ ℜ^*n*^. In the image denoising problem, the signal at each node (i.e. pixel) corresponds to the image intensity.

### Signal smoothness with respect to the intrinsic structure of graph

As is stated above, image denoising is an ill-posed problem, and thus prior knowledge about the sought image is required for the regularization. When an image is represented in the form of graph signal, the attribute that can describe graph signal should be chosen as the prior knowledge accordingly. In this paper, we take the graph signal smoothness as prior knowledge. Smoothness is one of the most important properties of graph signals and requires taking into consideration the intrinsic structure of the data domain. Here, the intrinsic structure refers to the weighted graph onto a sampled manifold [16]. For analyzing continuous signals on differentiable manifolds, discrete calculus provides the right tools to operate [17]. Discrete differential operators defined on a graph have been widely explored in the literature. Herein, we only examine some important concepts and definitions [18] in order to derive an accurate mathematical description of the smoothness of signal on a graph.

The edge derivative of a signal **f** along the edge *e* = (*i*, *j*) connecting the nodes *i* and *j* is defined as:

$${\frac{\partial f}{\partial e}|}_{i}=\sqrt{W_{i,j}}\left[f\left(j\right)-f\left(i\right)\right].$$ (4)

Based on the Eq (4), the graph gradient of **f** at node *i* is the vector:

$$\nabla_{i}f={\left\{{\frac{\partial f}{\partial e}|}_{i}\right\}}_{e\in \varepsilon \;\text{s}.\text{t}.\;e=(i,j)\;\text{for}\;\text{some}\;j\in V}.$$ (5)

The local variation at node *i* can be defined as:

$$\begin{array}{ll} {\left\|\nabla_{i}f\right\|}_{2} & ={\left[\sum_{e\in \varepsilon \;\text{s}.\text{t}.\;e=(i,j)\;\text{for}\;\text{some}\;j\in V}{\left({\frac{\partial f}{\partial e}|}_{i}\right)}^{2}\right]}^{\frac{1}{2}}\\ & ={\left[\sum_{j\in N_{i}}W_{i,j}{\left[f(i)-f(j)\right]}^{2}\right]}^{\frac{1}{2}} \end{array}.$$ (6)

This formula provides a measure of local smoothness of graph signal **f** around node *p*. The global smoothness using the discrete *p*-Dirichlet form of **f** is then defined as:

$$S_{p}(f)=\frac{1}{p}\sum_{i\in V}{\left\|\nabla_{i}f\right\|}_{2}^{p}=\frac{1}{p}{\sum_{i\in V}\left[\sum_{j\in N_{i}}W_{i,j}{\left[f\left(j\right)-f\left(i\right)\right]}^{2}\right]}^{\frac{p}{2}}.$$ (7)

### AGTV algorithm

AGTV algorithm was proposed for tomographic data reconstruction. The whole algorithm can be divided into five steps: (i) Project input data into the sinogram space to obtain a filtered back projection (FBP). (ii) Construct a patch graph from the FBP. (iii) Formulate an objective function that takes graph total variation and adjoint operator of the wavelet transform as regular terms. (iv) Solve the objective function with the forward–backward primal dual (FBPD) algorithm. (v) Repeat step (ii) to (iv) until convergence.

Although the AGTV algorithm can perform well on the tomographic data denoising, there still exist some shortages that hinder it from being applicable to the natural image denoising problem. First, only image intensity is taken into consideration during the graph construction while ignoring the patch location information. Second, the patch graph needs to be constructed repeatedly in the whole algorithm, which will lead to the tedious hyperparameter tuning problem. How to overcome both deficiencies and construct an effective GTV-based image denoising algorithm becomes our main motivation to propose the NPGTV algorithm.

## A non-local patch graph total variation

**Fig 1:**
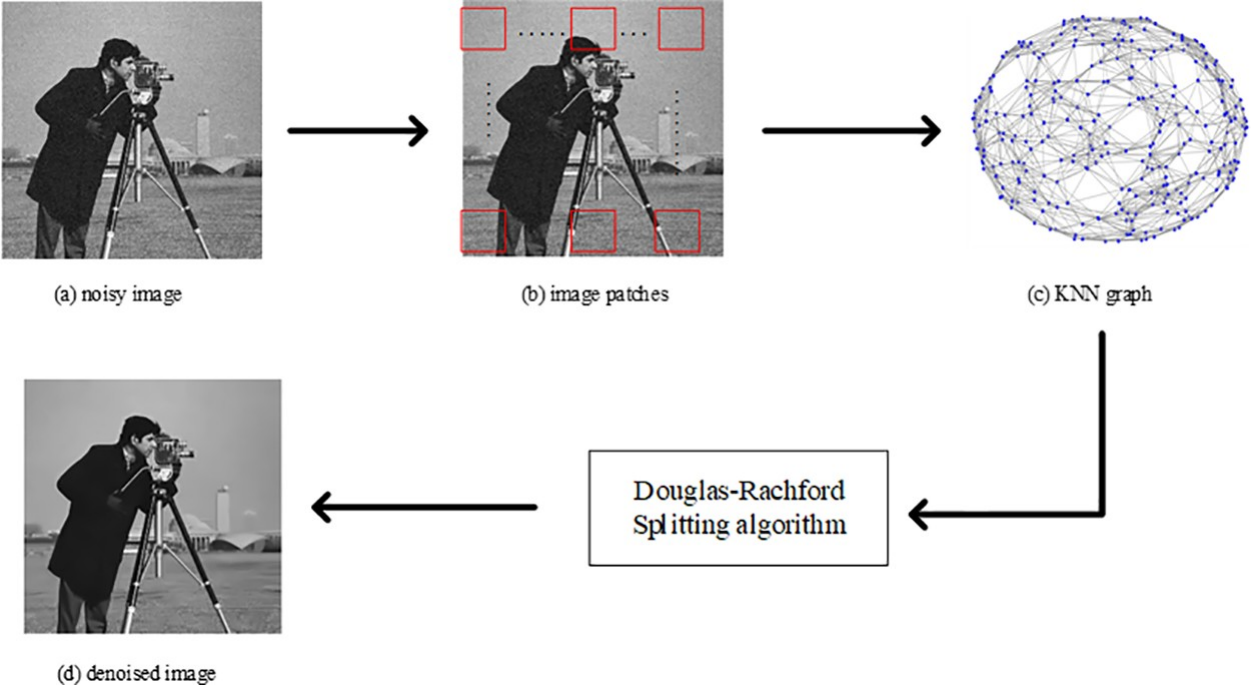
The procedure of our NPGTV method. The image is contaminated by the additive Gaussian noise with 10dB standard deviation. (The original Cameraman image in our paper is taken from Fig 1 in [19] published by PLOS one, which is licensed under the Creative Commons Attribution International License (CC BY 4.0). KNN graph is visualized through the Matlab software).

In this section, we describe the proposed NPGTV algorithm accordingly to three steps: (i) representation of an image as a weighted undirected graph; (ii) establishment and solving of the total variation model; (iii) description of our complete NPGTV proposal. This choice stands for the main steps of our algorithm depicted in Fig 1. First, as shown in Fig 1(a) and 1(b), a group of non-local image patches are extracted from a noisy image. By next, a KNN graph is derived from these patches as illustrated in Fig 1(c). Then the NPGTV model is established. Finally, the denoised image is achieved by performing the Douglas-Rachford splitting algorithm on to this model (Fig 1(d)).

### Total variation of graph signal

As we discuss above, Eq (7) can measure the graph signal smoothness. The *p* value in (7) can take 1, 2 and ∞. When *p* = 1, *S*_1_(**f**) is the total variation of the signal on a graph [20]:

$$\begin{array}{ll} S_{1}(f) & ={\left\|f\right\|}_{TV}=\sum_{i\in V}{\left\|\nabla_{i}f\right\|}_{2}={\sum_{i\in V}\left[\sum_{e\in \varepsilon \;\text{s}.\text{t}\;.\;e=(i,j)\;\text{for}\;\text{some}\;j\in V}{\left({\frac{\partial f}{\partial e}|}_{i}\right)}^{2}\right]}^{\frac{1}{2}}\\ & ={\sum_{i\in V}\left[\sum_{j\in N_{i}}W_{i,j}{\left[f\left(i\right)-f\left(j\right)\right]}^{2}\right]}^{\frac{1}{2}} \end{array}.$$ (8)

From (8), one can easily find that edge weights have an important effect on graph total variation. Thus, for a same image, changing the graph topology by modifying its edges will lead to different graph total variation.

Notice that in [21] another GTV based on the *l*_*p*_ norm was used:

$${\left\|f\right\|}_{TV}={\left\|f-\frac{1}{\left|\rho_{\text{max}}\left(A\right)\right|}Af\right\|}_{2}^{2},$$ (9)

where **A** stands for the adjacent matrix and *ρ*_max_ (**A**) denotes the eigenvalue with the largest magnitude. Although (9) can measure graph smoothness generally, it is not necessary vanished for constant graph signal and may be zero for non-constant signal as mentioned in [20]. As a consequence, in this paper, we limit our discussion to (8).

### Modified graph representation

**Fig 2:**
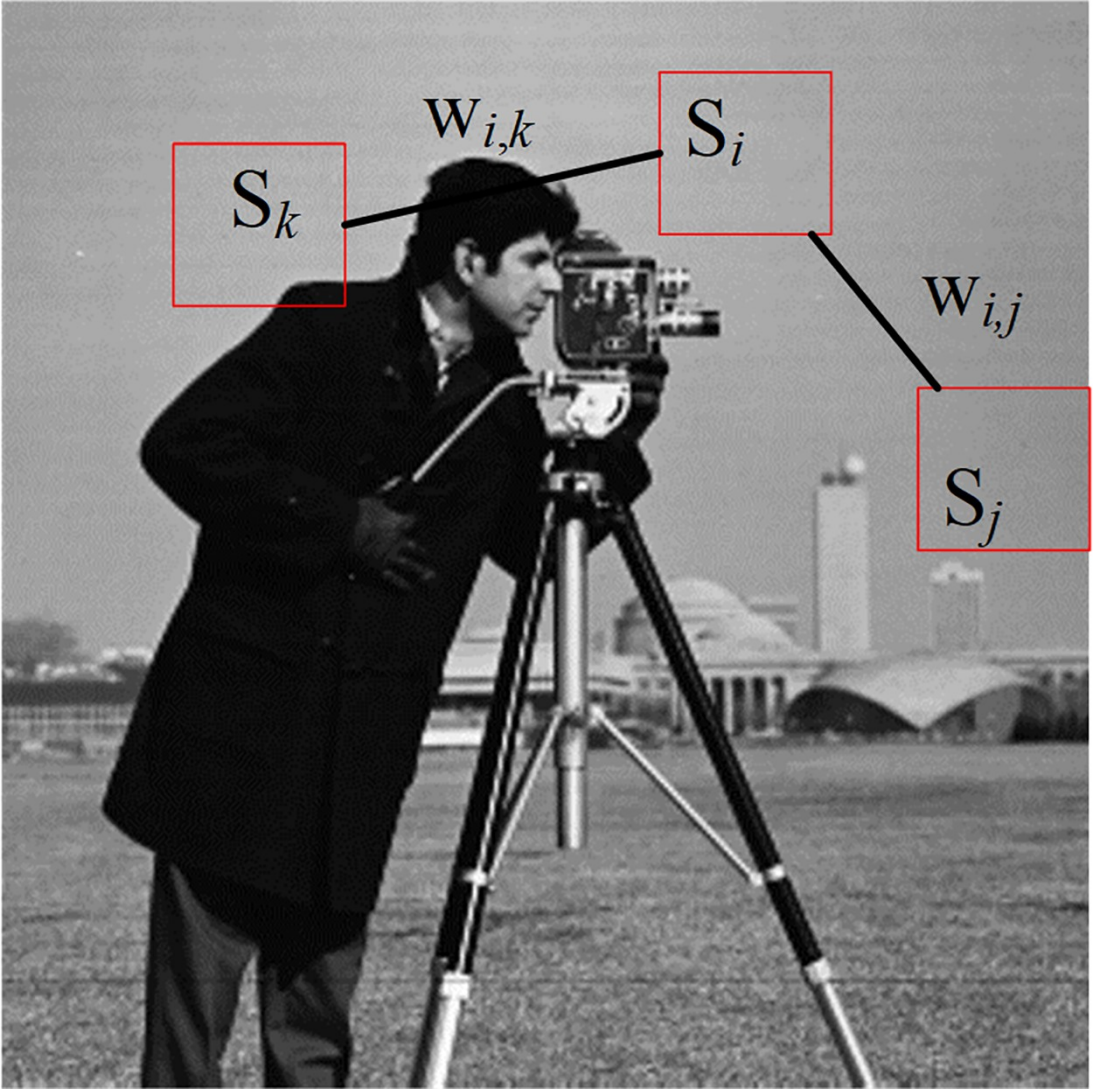
Examples of similarities between patches in the standard Cameraman image. S_*i*_, S_*j*_ and S_*k*_ are three distinct patches. *w*_*i*,*j*_ and *w*_*i*,*k*_ are weights on the edges e_*i*,*j*_ and e_*i*,*k*_ respectively.

One of the core steps of our method is to construct from an image a graph *G* for GTV regularization. To do so, we build a weighted undirected graph *G* = (*V*, *E*) to describe an image by considering its pixels as elements of *V*. The set *E* contains the corresponding edge information. The edge *e*_*m*,*n*_ only exists if the node *v*_*m*_ and *v*_*n*_ are connected. One naïve way consists in connecting each pixel to its neighbors. One can thus obtain a 4-connect graph or an 8-connect graph. Another strategy, like in AGTV [11], connects image patch center at each pixel through the *K* nearest neighbor (KNN) algorithm. However, both methods suffer from various drawbacks. The former, being a local model, will tend to alter image details when denoising. The latter is a non-local model that merely uses patch intensity to calculate the distance between two image patches without considering central pixel coordinate. To go beyond these disadvantages, we propose a graph construction method that combines patch intensity with pixel coordinates. The method consists of four steps:

1. The whole image *u* ∈ ℜ^*n*×*n*^ is first divided into a series of overlapped patches. Let us note *S*_*i*_ be a *s* × *s* image patch whose center is at the *i*th pixel;
2. Each patch is then vectorized and concatenated with its center pixel coordinates. More clearly a given image patch *S*_*i*_ is expanded into a vector **v**_*i*_ into which, the coordinates of the *i*th pixel (*i*_*row*_, *i*_*col*_) are incorporated so to get an access to a new vector ${v^{\prime }}_{i}=\left(v_{i},\lambda i_{row},\lambda i_{col}\right)$, where *λ* is a parameter that expresses the spatial constraint;
3. Each image patch is connected with its *k* nearest neighbors depending on the Euclidean distance metric through the KNN algorithm. In details, the Euclidian distance between two image patches *S*_*i*_ and *S*_*j*_ is such as $d\left(i,j\right)={\left\|{v^{\prime }}_{i}-{v^{\prime }}_{j}\right\|}_{2}$;
4. Finally, using the Gaussian kernel weighting scheme (10), the graph weight matrix *W* is computed as follows:
  $$w(i,j)=\{\begin{array}{ll} \text{exp}(-\frac{d^{2}(i,j)}{\sigma^{2}}) & \text{if}\;\text{patch}\;S_{i}\;\text{and}\;S_{j}\;\text{is}\;\text{connected}\\ 0 & \;\text{otherwise} \end{array},$$ (10)
  where *σ* is a parameter that controls the sensitivity of the similarity measure to the noise. This one is empirically fixed to 20% of the sum of the noise variance like in[22]. Such procedure is illustrated in Fig 2.

### Parameters analysis

**Fig 3:**
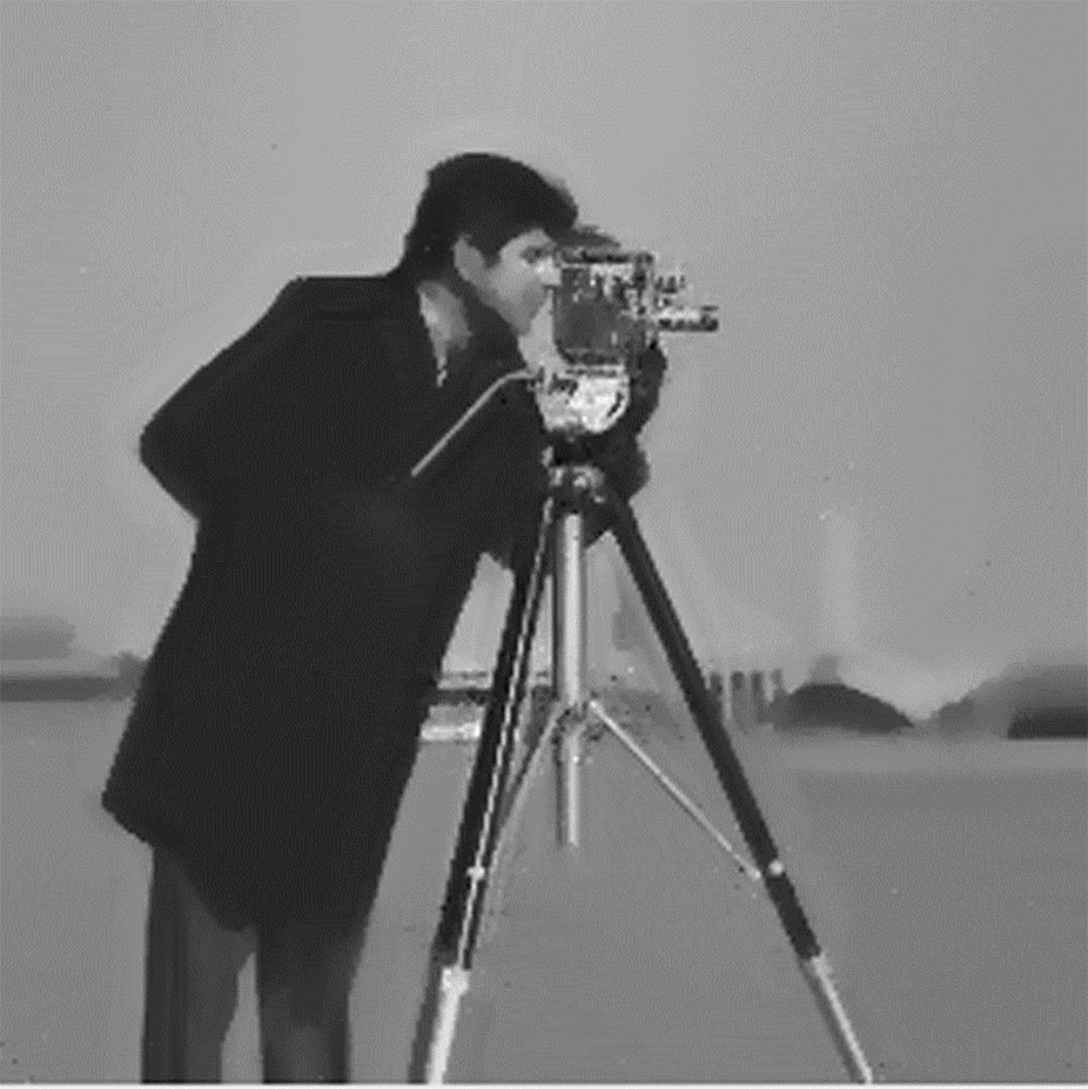
256×256 denoised Cameraman image under 30db noise. The patch size is 20, *K* is 9 and *λ* is 0.05. Although the noise is removed, some image details are also eliminated such as building outline and grass on the ground. Besides, the image is blurred. A virtual outline appears around the image edges.

From the above, it is easy to find that the patch size *s*, the *K* value of KNN algorithm and the spatial constraint parameter *λ* will have an impact on the denoising result. All three parameters are relevant to the noise level. Generally speaking, these parameters will take large values under high noise levels, and vice versa. But they cannot be too high. For the first two parameters, the larger the patch size and *K*, the smoother the image will be. If the patch size and *K* take too large values, some image details will be removed. Besides, too large patch size will lead to image edge blur (just as illustrated in Fig 3). Too large *K* values will also increase the calculation cost slowing down our method. Thus in the experiments presented in our paper, we set a patch size of 9×9 pixels under high noise level (larger than 15db) and 5×5 pixels under weak noise level (less than15db). The value of *K* is fixed to 5. Such parameters have been shown to be robust while details and fine structure can be better preserved.

**Fig 4:**
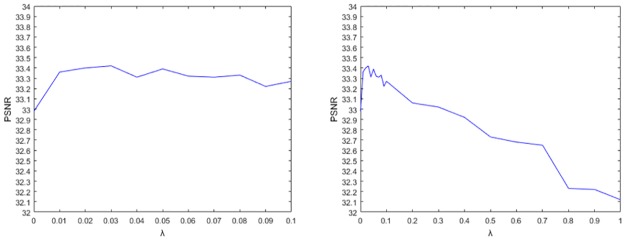
PSNR values of the denoised Cameraman image in the case of 10db noise with various *λ*. The image size is 256×256 pixels. In all experiments, patch size is 5×5 pixels and *K* is fixed to 5. Left: Obtained PSNR values for *λ* values between 0 and 0.1. Right: PSNR values for *λ* values in the range [0,1]. It is easy to observe that the relation between *λ* and the performance of the NPGTV algorithm is consistent with our theoretical analysis.

As for the spatial constraint parameter *λ*, the location information of the patch is normalized into the range of gray level of the image. By doing so, patch intensity and location are more relevant when constructing the graph with the KNN algorithm. Notice that, when *λ* = 0, ${v^{\prime }}_{i}$ is degraded into *v*_*i*_. As a consequence, the similarity between two patches is measured based on the distance of their intensity level. When *λ* takes a high value, patch location will become the main ingredient to determine the similarity between two patches. Taking such value may not work well since it is extremely sensitive to minor transformations, both in geometry (shifts and rotations) and in imaging conditions (lighting or noise) [23]. Therefore, the selection of the value of *λ* heavily depends on the range of gray level of the image. In our experiment, the gray level is normalized between 0 to 1 considering images of size 512×512 or 256×256 pixels. *λ* should guarantee that the values of *λi*_*row*_ and *λi*_*row*_ not too far from this range. Thus, we set *λ* in the range between 0.01 and 0.1. Fig 4 well validates the effectiveness of our strategy to select the value of *λ*.

### Optimization solution of NPGTV

After image graph representation is obtained, combining with the definition of graph total variation in (8), we construct the NPGTV model and solve it. We set **u**_0_ the graph signal derived from the original noisy image and **u** the corresponding recovered one, i.e. the original image. Our model can be formulated as a convex optimization problem as follows:

$$\underset{u}{\text{arg}\;\text{min}}{\left\|u\right\|}_{\text{TV}}\;s.t.{\left\|u-u_{0}\right\|}_{2}\leq \varepsilon ,$$ (11)

where *ε* is the radius of a L_2_ ball. In this paper, the operator splitting method, one of the most important techniques, is employed to solve the model (11). The basic idea of the method is to divide the optimized object into several convex functional in the form of summation. Thus, a complicated problem can be decomposed into several subproblems that are easier to solve. For (11), we set *f*_1_(**u**) = ||**u**||_TV_ and *f*_2_ as the indicator function of the set **H** defined as ||**u** − **u**_0_|| ≤ *ε*. Then the prox of *f*_1_ and *f*_2_ can be acquired through:

$$prox_{f_{1},\gamma }\left(u_{0}\right)=\underset{u}{\text{arg}\;\text{min}}\frac{1}{2}{\left\|u-u_{0}\right\|}_{2}^{2}+\gamma {\left\|u\right\|}_{\text{TV}},$$ (12)

$$prox_{f_{2},\gamma }\left(u_{0}\right)=\underset{u}{\text{arg}\;\text{min}}\frac{1}{2}{\left\|u-u_{0}\right\|}_{2}^{2}+i_{H}\left(u\right),$$ (13)

where *i*_*H*_ (**u**) is zero if **u** is in the set **H** and infinity otherwise. *prox*_*f*_: ℜ^*n*^ → ℜ^*n*^ is the proximal operator which is defined as follow [24]:

$$prox_{f}\left(x\right)=\underset{y}{\text{arg}\;\text{min}}\;\frac{1}{2}{\left\|x-y\right\|}_{2}^{2}+f\left(y\right),$$ (14)

The function on the right-hand in (14) is strongly convex and not every infinite, so it has a unique minimizer for every *x* ∈ ℜ^*n*^. Eqs (12) and (13) can be solved by various approximate splitting algorithms, such as Forward-Backward Splitting (FBS) [25], Douglas-Rachford Splitting (DRS) [26] or Alternating direction method (ADM) [27], and Primal-Dual hybrid gradient (PDHG) [28]. The convergence conditions required by Douglas-Rachford splitting (DRS) are slack. Besides, it has more general convergence character when solving the finite-dimensional problem with the fixed-step DRS iterative scheme. More important, it does not need the decomposed subproblems to be differentiable like for the FBS algorithm [29]. Therefore, in our paper, we choose the DRS algorithm to solve the NPGTV model. Applications of the DRS algorithm in signal and image processing can be found in[30–33].

DRS algorithm was originally used to solve the equation *μ* = **Ax** + **Bx**, where **A** and **B** are both positive definite matrices. Later, it was used to solve non-linear problems. For any *γ* > 0, there exists at least one solution for the unconstrained non-convex problem shown in (15)

$$\underset{x\in {ℜ}^{N}}{\text{min}}\;f_{1}\left(x\right)+f_{2}\left(x\right),$$ (15)

This solution satisfies the following two conditions:

$$\left\{ \begin{array}{l} x=prox_{\gamma ,f_{2}}y\\ prox_{\gamma ,f_{2}}y=prox_{\gamma ,f_{1}}\left(2-prox_{\gamma ,f_{2}}y-y\right) \end{array}\right..$$ (16)

with *prox*_*γ*,*f*_ *y* expressed as [24]:

$$prox_{\gamma ,f}y=\underset{y}{\text{arg}\;\text{min}}\;\frac{1}{2\gamma }{\left\|y-x\right\|}_{2}^{2}+f\left(y\right),$$ (17)

where *γ* is a parameter. Algorithm 1 presents the derivation process of the DRS algorithm for NPGTV. Here the graph signal **u**_0_ derived from the original noisy image is set as the algorithm initial input. *λ*_*n*_ stands for the iterative step. *tol* denotes the stopping tolerance parameter. The detailed derivation process of the algorithm can be found in [34].

**Algorithm 1**: Douglas-Rachford Splitting algorithm for NPGTV

INPUT: *y*_0_ = **u**_0_, *γ* > 0, *ε* ∈[0,1], *tol* > 0

ITERATIVELY: **for** n = 0, 1, ….*I*-1 **do**

  $x_{n}=prox_{\gamma ,f_{2}}y_{n}$

  *λ*_n_ ∈ [*ε*, 2 − *ε*]

  $y_{n+1}=y_{n}+\lambda_{n}\left(prox_{\gamma ,f_{1}}y_{n}\left(2x_{n}-y_{n}\right)-x_{n}\right)$

  **if** $\frac{y_{n+1}-y_{n}}{y_{n+1}}<tol$ **then**

  BREAK

  **end if**

 **end for**

OUTPUT: **u** = *y*_*n*+*l*_

The whole procedure of our NPGTV algorithm is summed-up in Algorithm 2.

**Algorithm 2**: Non-local patch graph total variation

Input: Noisy grayscale image

Processing steps:

 A. Transform the noisy image into a modified non-local patch graph structure *G*;

 B. According to Eq (10) calculate the edge weights;

 C. Calculate Graph Total Variation according to Eq (8);

 D. Construct the optimization form of the denoising problem of total variation on graph according to Eq (11);

 E. Solve the NPGTV model by means of the DRS algorithm as Algorithm 1.

Output: The denoised image

### Algorithm complexity analysis

The computational complexity of the NPGTV algorithm depends on the KNN graph construction and on the optimization algorithm given in Algorithm 1. For an image of *n* pixels, *n* overlapped patches can be achieved. The computational complexity when directly building the graph with KNN algorithm is *O*(*n*^2^). In order to reduce such complexity, and inspired by the AGTV algorithm, we decided to use the open-sourced library called FLANN [35]. Set the size of each image patch is *s* × *s* pixels and *K* value in KNN algorithm is fixed. With FLANN, the computational complexity of the graph construction is reduced to *O*(*n*log(*n*)). Let *I* be the maximum number of iterations for the DRS algorithm to converge. In that case, the DRS computational cost is *O*(*I*|*E*|), where |*E*| represents the number of edges in the graph *G* = (*V*, *E*). As *G* is a KNN graph where *E* ≈ *Kn*, the computational complexity of our optimization algorithm is linear in the size of the number of the graph vertex *n*, i.e., *O*(*IKn*). Based on the above analysis, the whole complexity of the NPGTV algorithm is *O*(*IKn* + *n*log(*n*)).

## Experiments

This section presents the experimental results we obtained by applying our method to the classic benchmark images considered in the literature. Performances of our scheme are also compared to several state-of-the-art denoising methods. Tests have been conducted using Graph Signal Processing MATLAB toolbox (GSPBox) on a PC with an Intel 4.0 GHz CPU and 16 GB of memory.

**Fig 5:**
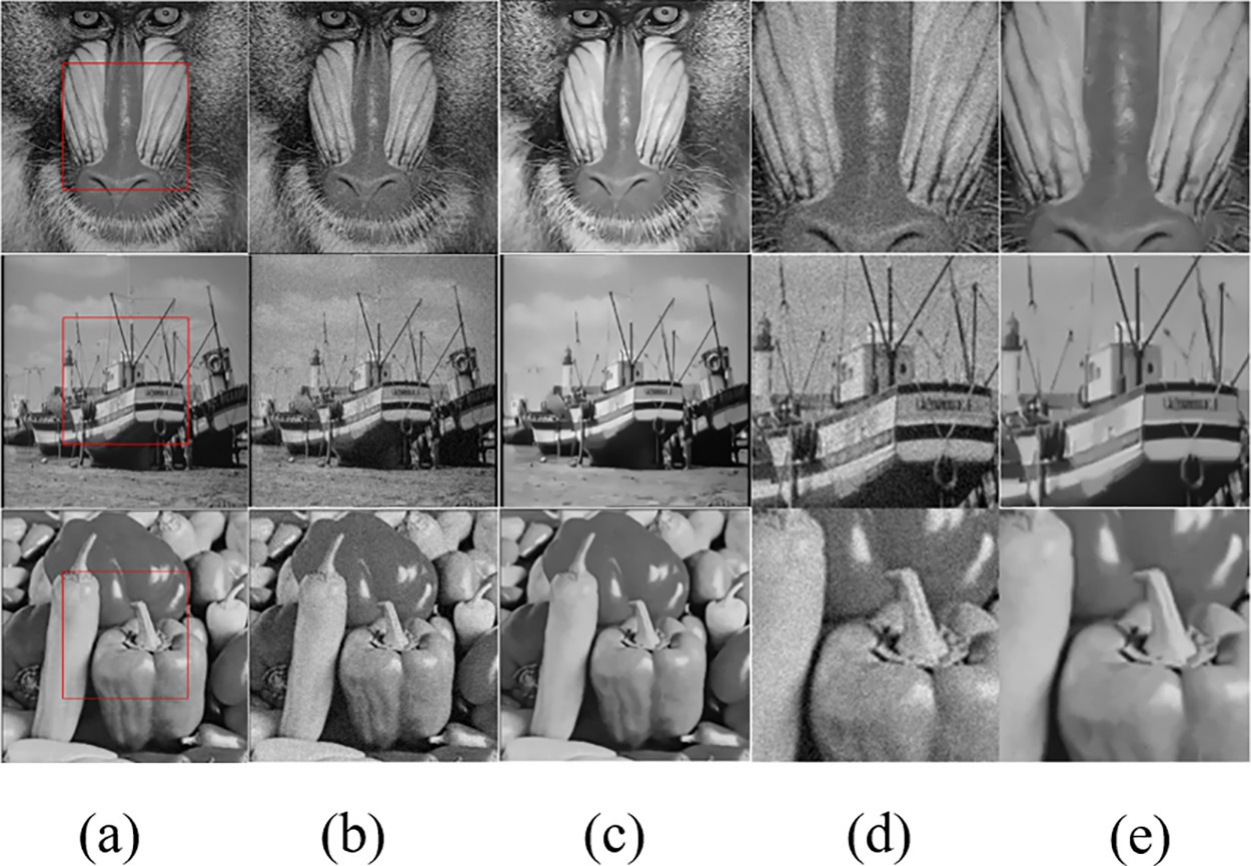
Results of the proposed NPGTV algorithm on different test images; images of reference in the literature. Column (a): Original images: Baboon, Boat and Pepper. (b) Images are corrupted with the noise at *σ* = 10dB. (c) Denoising results. All three examples are filtered using a patch size of 5×5, *K* = 5. (d) Zoomed regions from noisy images and (e) the corresponding denoised image regions. (The original Baboon and Boat images in our paper are taken from Fig 7 in [36] published by the journal Mathematical Problems in Engineering, and Pepper image in our paper is taken from Fig 1 in [19] published by the journal PLOS one. Both journals are licensed under the Creative Commons Attribution International License (CC BY 4.0)).

In the following examples, images were contaminated by additive independent and identically distributed zero-mean Gaussian noise (AIIDZMGN) with the standard deviation *σ*. To investigate the effect of the denoising process, as in [1] [4] and [8], we choose the standard Baboon, Boat and Pepper images. The top row of Fig 4 highlights the denoising results obtained on the standard Baboon image with NPGTV considered an AIID zero-mean Gaussian noise of 10 dB. Zoomed medallions in Fig 5 columns (d) and (e) allow a better screening of the result. We can see that an effective suppression of noise is achieved while complicated skin textures and periodic patterns are preserved. Similar comments can be made on the Boat and Pepper images (middle and bottom Fig 5 rows, respectively). Such qualitatively good performance can be explained by the presence of similar textures not only in the immediate neighborhood of a given pixel but also of distant pixels.

**Fig 6:**
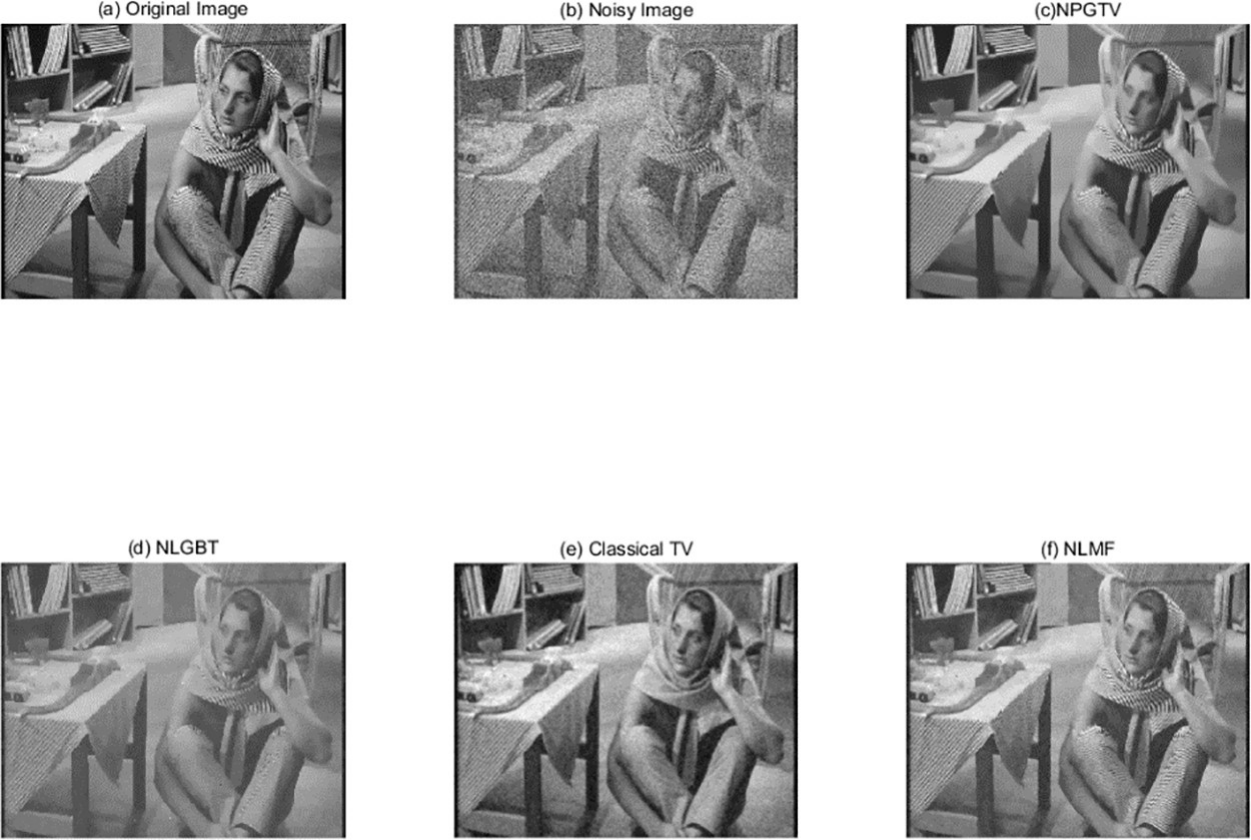
Results of different denoising methods on 576×720 Barbara image. The image is corrupted by the noise at σ = 30 dB. (a): Original image. (b): Noisy image. (c): NPGTV with k = 5, λ = 0.05, patch size 9×9. (d): NLGTV algorithm. (e): Classical TV. (f) NLMF with search window size 3×3, similar widow size 5×5 and the standard deviation is set to 0.1. (The original Barbara image in our paper is taken from Fig 1 in [19] published by the journal PLOS one, which is licensed under the Creative Commons Attribution International License (CC BY 4.0)).

Comparisons with some state-of-the-art denoising methods have been carried out for various levels of noise. These state-out-of-art method set includes the non-local graph based transform (NLGBT) method [22], non-local means (NLM) filter scheme [4], and the classical total-variation approach (TV) [37]. In order to make a fair assessment, we manually choose the parameters’ values of each method in order to obtain the best results they can provide for a given image and noise level. Fig 6 displays the results obtained for the Barbara image contaminated by an additive zero-mean Gaussian noise of 30dB. (More experiment results of some other standard images can be founded in Figs A-E of S1 Appendix) In order to quantitatively evaluate and compare these methods, we use Peak Signal to Noise Ratio (PSNR) and structural similarity index (SSIM) [38]. Considering images of *m*×*l* pixels, they are defined as:

$$\begin{array}{ll} PSNR & =10\text{log}\left(\frac{\sum_{i=1}^{m}\sum_{j=1}^{l}{\left(255\right)}^{2}}{\sum_{i=1}^{m}\sum_{j=1}^{l}{\left(u\left(x,y\right)-\hat{u}\left(x,y\right)\right)}^{2}}\right)\\ & =10\cdot {\text{log}}_{10}\left[\frac{255^{2}}{MSE}\right] \end{array}$$ (18)

$$SSIM\left(u,\hat{u}\right)=\frac{\left(2\mu_{u}\mu_{\hat{u}}+c_{1}\right)\left(2\sigma_{u\hat{u}}+c_{2}\right)}{\left(\mu_{u}^{2}+\mu_{\hat{u}}^{2}+c_{1}\right)\left(\sigma_{u}^{2}+\sigma_{\hat{u}}^{2}+c_{2}\right)}.$$ (19)

where *u* and $\hat{u}$ denote the original image and its corresponding denoised version, respectively; *μ*_*u*_ and $\mu_{\hat{u}}$ stand for the average of *u* and $\hat{u}$ while *σ*_*u*_ and $\sigma_{\hat{u}}$ represent their standard deviation; $\sigma_{u\hat{u}}$ means the covariance of *u* and $\hat{u}$; *c*_1_ and *c*_*2*_ are constant values to avoid denominator be zero.

The PSNR and SSIM measures we obtained are reported in Table 1. In both cases, our NPGTV method provides noticeably better results for both noise elimination and feature preservation than the classical TV algorithm and the low-pass filtering on graph method. The proposed NPGTV algorithm not only achieves better performance than the NLGBT algorithm, but also needs less time than the NLGBT algorithm. In addition, the TV method eliminates some details that are preserved by NPGTV like the details and textures of the Barbara image.

**Table 1:** Obtained PSNR values and SSIM values for different denoising methods on some benchmark images. In each cell, four results are represented-Top left: NPGTV. Top right: NGBT. Bottom left: TV. Bottom right: NLMF. The best result among four is highlighted in bold in each cell.

Image	$\left[\begin{array}{cc} \text{NPGTV} & \text{NGBT}\\ \text{TV} & \text{NLMF} \end{array}\right]$				Noise standard deviation					
	10		15		20		25		30	
*Cameraman*	**33.42**	32.26	**30.41**	30.04	**29.52**	28.49	**28.5**	27.14	**27.64**	26.33
	**0.9151**	0.8897	**0.8537**	0.8451	**0.8416**	0.7957	**0.8184**	0.7208	**0.7938**	0.6806
	23.27	29.45	23.22	29.3	23.15	29.04	23.06	28.41	22.94	27.41
	0.7416	0.8349	0.7309	0.8336	0.7133	0.8229	0.6941	0.7861	0.6715	0.7091
*Lena*	**34.88**	33.63	**32.93**	31.79	**31.48**	30.25	30.36	29.3	**29.44**	28.66
	**0.8946**	0.8773	**0.8591**	0.8480	**0.8371**	0.8185	**0.8111**	0.7982	**0.7852**	0.7833
	29.35	32.07	29.17	31.90	28.90	31.45	28.58	**30.61**	28.21	29.25
	0.8286	0.8496	0.8177	0.8484	0.8021	0.8352	0.7850	0.7949	0.7627	0.7163
*Barbara*	**32.94**	31.85	**30.93**	29.46	**29.42**	27.45	**28.97**	26.2	**27.80**	25.22
	**0.9130**	0.8983	**0.8828**	0.8491	**0.8502**	0.7927	**0.834**	0.7482	**0.7993**	0.7090
	24.25	29.93	24.19	29.75	24.10	29.37	23.98	28.66	23.86	27.62
	0.6959	0.8552	0.6881	0.8538	0.6774	0.8433	0.6636	0.8111	0.6488	0.7523
*House*	**35.02**	34.52	**33.59**	32.84	**32.24**	31.43	**31.36**	30.29	**30.52**	29.35
	**0.8801**	0.8716	**0.8596**	0.8537	**0.8312**	0.8400	**0.8223**	0.8244	**0.8206**	0.8091
	27.40	32.83	27.25	32.42	27.11	31.99	26.86	30.99	26.59	29.4
	0.8076	0.8528	0.7946	0.8502	0.7808	0.8395	0.7645	0.8004	0.7408	0.7133
*Bacteria*	**36.97**	34.61	34.60	32.40	33.63	30.33	**32.65**	29.70	31.22	29.11
	**0.9612**	0.9509	0.9539	0.9336	**0.9388**	0.9104	**0.9276**	0.9034	**0.9281**	0.8934
	32.95	35.85	32.57	**35.08**	31.88	**33.90**	31.36	32.42	**31.36**	30.40
	0.9597	0.9645	0.9492	**0.9574**	0.9347	0.9375	0.9166	0.8931	0.8989	0.8111
*Moon*	**36.96**	35.18	**35.23**	33.76	34.29	32.78	**33.29**	32.14	**32.90**	31.58
	**0.8851**	0.6156	**0.8583**	0.5656	**0.8469**	0.5495	**0.8267**	0.5409	**0.8208**	0.5345
	33.23	35.46	32.74	35.10	32.16	**34.46**	31.59	33.19	30.90	31.15
	0.8650	0.8775	0.8288	0.8581	0.7943	0.8305	0.7644	0.7838	0.7336	0.6998

**Fig 7:**
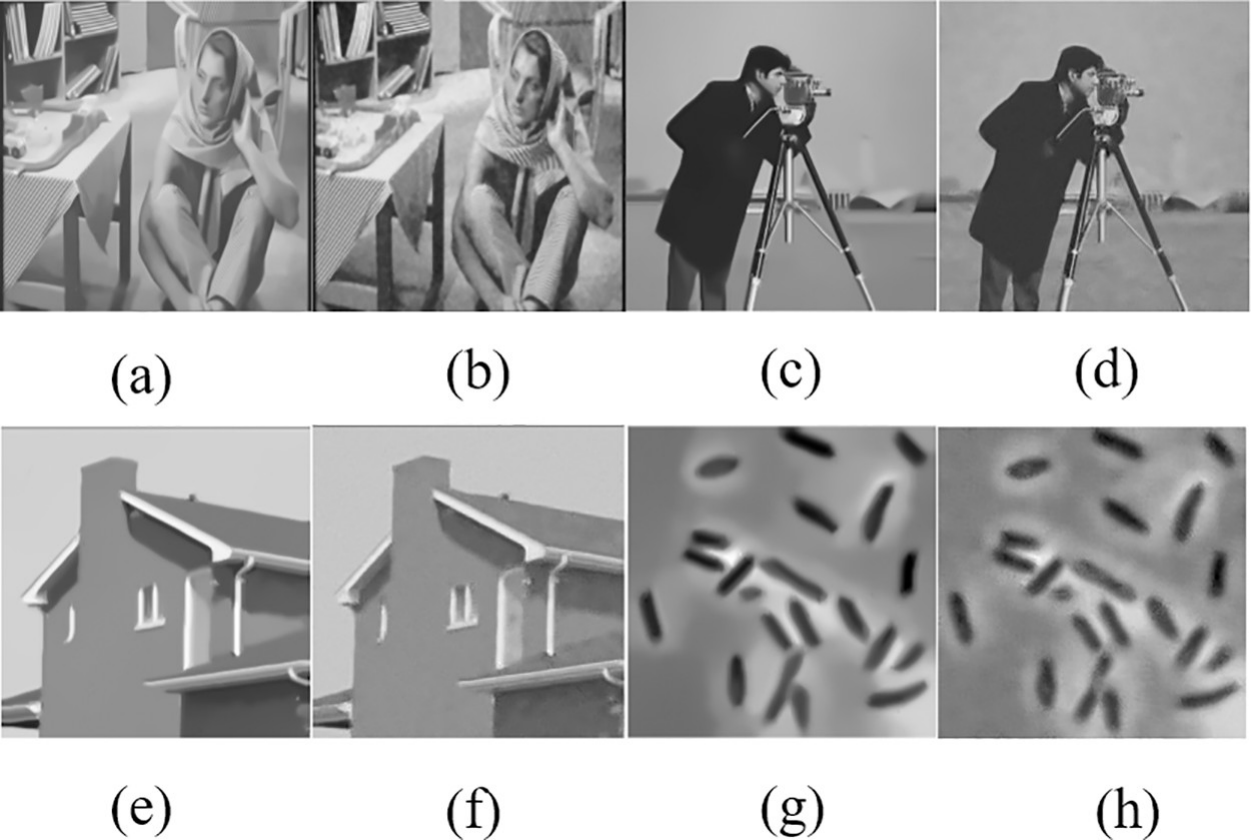
Comparison between the proposed NPGTV algorithm and AGTV on some benchmark images which are contaminated by the noise at *σ* = 30 dB. a, c, e, and g are the denoised images obtained with the proposed NPGTV solution while the rest of images are the results achieved with AGTV. (The original House image in our paper is taken from Fig 1 in [19] published by the journal PLOS one. The original Bacteria image is taken from Fig 5 in [39] published by the journal World Journal of Engineering and Technology. Both journals are licensed under the Creative Commons Attribution International License (CC BY 4.0)).

Although our method has a close link with the AGTV algorithm[11], it is far better than AGTV in terms of complexity and denoising effect. In Fig 7, we compare our method and AGTV on some benchmark images. These images are corrupted by the noise at *σ* = 30 dB. Intuitively, the images denoised with our method preserve more image details compared to AGTV. Table 2 shows that our method achieves higher PSNR and SSIM values on the most testing images.

**Table 2:** PSNR and SSIM for NPGTV and AGTV on some benchmark images.

Image	[NPGTV AGTV]				Noise stand variation					
	10		15		20		25		30	
*Cameraman*	**33.42**	29.64	**30.41**	28.69	**29.52**	27.72	**28.5**	26.69	**27.64**	26.20
	**0.9151**	0.8444	**0.8537**	0.8329	**0.8416**	0.8166	**0.8184**	0.7953	**0.7938**	0.7235
*Lena*	**34.88**	33.11	**32.93**	31.37	**31.48**	29.59	**30.36**	29.05	**29.3**	28.64
	**0.8946**	0.8692	**0.8591**	0.8483	**0.8371**	0.8177	**0.8111**	0.7734	**0.7814**	0.749
*Barbara*	**32.94**	30.28	**30.93**	28.51	**29.42**	27.67	**28.97**	26.66	**27.8**	26.15
	**0.9130**	0.8702	**0.8828**	0.8184	**0.8502**	0.7905	**0.834**	0.7531	**0.7993**	0.7315
*House*	**35.02**	34.21	**33.59**	33.10	**32.24**	31.69	**31.36**	30.33	**30.52**	29.77
	**0.8801**	0.8831	**0.8596**	0.8554	**0.8312**	0.8353	**0.8223**	0.8118	**0.8206**	0.784
*Bacteria*	**36.97**	35.25	**34.60**	33.31	**33.63**	31.59	**32.65**	30.06	**30.32**	29.85
	**0.9612**	0.9644	**0.9539**	0.9483	**0.9388**	0.9300	**0.9276**	0.9056	**0.9104**	0.887
*Moon*	**36.96**	36.09	**35.23**	34.78	**34.29**	34.00	33.29	**33.31**	**32.95**	31.88
	**0.8851**	0.8780	**0.8583**	0.8559	**0.8469**	0.8342	**0.8267**	0.8219	0.8208	**0.8329**

## Conclusion

In this paper, we have proposed a non-local patch graph total variation algorithm for image denoising. Unlike most continuous Total Variation-based methods for image denoising, the problem has been formulated using a graph-spectral approach. An imagewas represented as an undirected graph whose edge weights are computed by means of a Gaussian kernel function, where Euclidean distance is calculated with two modified image patches instead of two isolated pixels. The numerical implementation of the algorithm was performed through the Douglas-Rachford Splitting algorithm. We also have demonstrated the relationships between our graph denoising method and a number of alternatives including the classical total variation and adaptive graph total variation. Qualitative and quantitative assessments, as well as the comparison of our approach with several state-of-the-art denoising methods, demonstrate its effectiveness and better behavior in general. Future works will focus on convex optimization—it could be improved—and extending our approach to some other kind images, such as color image etc. Besides, we plan to bring in some technologies like superpixel/supervoxel based methods [40, 41] to reduce the complexity of the graph construction.

## Supporting information

S1 Appendix This appendix contains Figs A-E. (PDF)

## References

[1] Milanfar P. A tour of modern image filtering: New insights and methods, both practical and theoretical. IEEE Signal Process Mag. 2013;30: 106–128.

[2] Karahanoglu FI, Bayram I, Van De Ville D. A signal processing approach to generalized 1-D total variation. IEEE Trans Signal Process. 2011;59: 5265–5274.

[3] Kheradmand A, Milanfar P. A general framework for kernel similarity-based image denoising. Global Conference on Signal and Information Processing (GlobalSIP), 2013 IEEE. pp. 415–418.

[4] Buades A, Coll B, Morel J-M. A non-local algorithm for image denoising. Computer Vision and Pattern Recognition, CVPR 2005 IEEE Computer Society Conference on. IEEE; 2005. pp. 60–65.

[5] Rudin LI, Osher S, Fatemi E. Nonlinear total variation based noise removal algorithms. Phys D Nonlinear Phenom. 1992;60: 259–268.

[6] Buades A, Coll B, Morel J-M. A review of image denoising algorithms, with a new one. Multiscale Model Simul. 2005;4: 490–530.

[7] Easley GR, Labate D, Colonna F. Shearlet-based total variation diffusion for denoising. IEEE Trans Image Process. 2009;18: 260–268. 10.1109/TIP.2008.2008070 19095539

[8] Zhang F, Hancock ER. Graph spectral image smoothing using the heat kernel. Pattern Recognit. 2008;41: 3328–3342.

[9] Sandryhaila A, Moura JMF. Discrete signal processing on graphs. IEEE Trans signal Process. 2013;61: 1644–1656.

[10] Pang J, Cheung G, Hu W, Au OC. Redefining self-similarity in natural images for denoising using graph signal gradient. Asia-Pacific Signal and Information Processing Association, 2014 Annual Summit and Conference (APSIPA). IEEE; 2014. pp. 1–8.

[11] Mahmood F, Shahid N, Skoglund U, Vandergheynst P. Adaptive graph-based total variation for tomographic reconstructions. IEEE Signal Process Lett. 2018;25: 700–704.

[12] You Y-L, Xu W, Tannenbaum A, Kaveh M. Behavioral analysis of anisotropic diffusion in image processing. IEEE Trans Image Process. 1996;5: 1539–1553. 10.1109/83.541424 18290071

[13] Smolka B, Wojciechowski KW. Random walk approach to image enhancement. Signal Processing. 2001;81: 465–482.

[14] Black MJ, Sapiro G, Marimont DH, Heeger D. Robust anisotropic diffusion. IEEE Trans image Process. 1998;7: 421–432. 10.1109/83.661192 18276262

[15] Tomasi C, Manduchi R. Bilateral filtering for gray and color images. Computer Vision, 1998 Sixth International Conference on. IEEE; 1998. pp. 839–846.

[16] Belkin M, Niyogi P. Towards a theoretical foundation for Laplacian-based manifold methods COLT. Springer; 2005 pp. 486–500.

[17] Grady LJ, Polimeni JR. Discrete calculus: Applied analysis on graphs for computational science. Springer Science & Business Media; 2010.

[18] Shuman DI, Narang SK, Frossard P, Ortega A, Vandergheynst P. The emerging field of signal processing on graphs: Extending high-dimensional data analysis to networks and other irregular domains. IEEE Signal Process Mag. 2013;30: 83–98.

[19] Liu G, Huang T-Z, Liu J, Lv X-G. Total variation with overlapping group sparsity for image deblurring under impulse noise. PLoS One. 2015;10: e0122562 10.1371/journal.pone.0122562 25874860 PMC4398568

[20] Berger P, Hannak G, Matz G. Graph signal recovery via primal-dual algorithms for total variation minimization. IEEE J Sel Top Signal Process. 2017;11: 842–855.

[21] Chen S, Sandryhaila A, Moura JMF, Kovačević J. Signal recovery on graphs: Variation minimization. IEEE Trans Signal Process. 2015;63: 4609–4624.

[22] Hu W, Li X, Cheung G, Au O. Depth map denoising using graph-based transform and group sparsity. Multimedia Signal Processing (MMSP), 2013 IEEE 15th International Workshop on. IEEE; 2013. pp. 1–6.

[23] Shakhnarovich G. Learning task-specific similarity. Massachusetts Institute of Technology; 2005.

[24] Parikh N, Boyd S. Proximal algorithms. Found Trends^®^ Optim. 2014;1: 127–239.

[25] Combettes PL, Wajs VR. Signal recovery by proximal forward-backward splitting. Multiscale Model Simul. 2005;4: 1168–1200.

[26] Combettes PL, Pesquet J-C. A Douglas–Rachford splitting approach to nonsmooth convex variational signal recovery. IEEE J Sel Top Signal Process. 2007;1: 564–574.

[27] Gabay D, Mercier B. A dual algorithm for the solution of nonlinear variational problems via finite element approximation. Comput Math with Appl. 1976;2: 17–40.

[28] Zhu M, Chan T. An efficient primal-dual hybrid gradient algorithm for total variation image restoration. UCLA CAM Rep. 2008;34.

[29] Combettes PL, Pesquet J-C. Proximal splitting methods in signal processing Fixed-point algorithms for inverse problems in science and engineering. Springer; 2011 pp. 185–212.

[30] Chaux C, Pesquet J-C, Pustelnik N. Nested iterative algorithms for convex constrained image recovery problems. SIAM J Imaging Sci. 2009;2: 730–762.

[31] Dupé F-X, Fadili JM, Starck J-L. A proximal iteration for deconvolving Poisson noisy images using sparse representations. IEEE Trans Image Process. 2009;18: 310–321. 10.1109/TIP.2008.2008223 19131301

[32] Durand S, Fadili J, Nikolova M. Multiplicative noise removal using L1 fidelity on frame coefficients. J Math Imaging Vis. 2010;36: 201–226.

[33] Setzer S, Steidl G, Teuber T. Deblurring Poissonian images by split Bregman techniques. J Vis Commun Image Represent. 2010;21: 193–199.

[34] Bot RI, Hendrich C. A Douglas—Rachford type primal-dual method for solving inclusions with mixtures of composite and parallel-sum type monotone operators. SIAM J Optim. 2013;23: 2541–2565.

[35] Muja M, Lowe DG. Scalable nearest neighbor algorithms for high dimensional data. IEEE Trans Pattern Anal Mach Intell. 2014;36: 2227–2240. 10.1109/TPAMI.2014.2321376 26353063

[36] Juang Y-S, Ko L-T, Chen J-E, Shieh Y-S, Sung T-Y, Hsin HC. Histogram modification and wavelet transform for high performance watermarking. Math Probl Eng. 2012;2012.

[37] Vogel CR, Oman ME. Iterative methods for total variation denoising. SIAM J Sci Comput. 1996;17: 227–238.

[38] Wang Z, Bovik AC, Sheikh HR, Simoncelli EP. Image quality assessment: from error visibility to structural similarity. IEEE Trans image Process. 2004;13: 600–612. 10.1109/tip.2003.819861 15376593

[39] Cao X, Miao J, Xiao Y. Medical image segmentation of improved genetic algorithm research based on dictionary learning. World J Eng Technol. 2017;5: 90–96.

[40] Kong Y, Deng Y, Dai Q. Discriminative clustering and feature selection for brain MRI segmentation. IEEE Signal Process Lett. 2014;22: 573–577.

[41] Kong Y, Wu J, Yang G, Zuo Y, Chen Y, Shu H, et al Iterative spatial fuzzy clustering for 3D brain magnetic resonance image supervoxel segmentation. J Neurosci Methods. 2019;311: 17–27. 10.1016/j.jneumeth.2018.10.007 30315839

