# Supplementary material for: Image denoising via a non-local patch graph total variation
Source: PLoS One. 2019 Dec 12;14(12):e0226067. doi: 10.1371/journal.pone.0226067 (PMC6907757; doi:10.1371/journal.pone.0226067)
Supplement: S1 Appendix — (PDF) [file pone.0226067.s001.pdf]

***PLoS One* Supporting Information Appendix S1**

Article title: Image denoising via a non-local patch graph total variation

Authors: Yan Zhang, Jiasong Wu, Youyong Kong, Gouenou Coatrieux, Huazhong Shu

The following Supporting Information is available for this article:

S1 Fig. A. Results of different denoising methods on Lena image.

S1 Fig. B. Results of different denoising methods on Cameraman image.

S1 Fig. C. Results of different denoising methods on House image.

S1 Fig. D. Results of different denoising methods on Bacteria image.

S1 Fig. E. Results of different denoising methods on Moon image.

Supplementary Figures

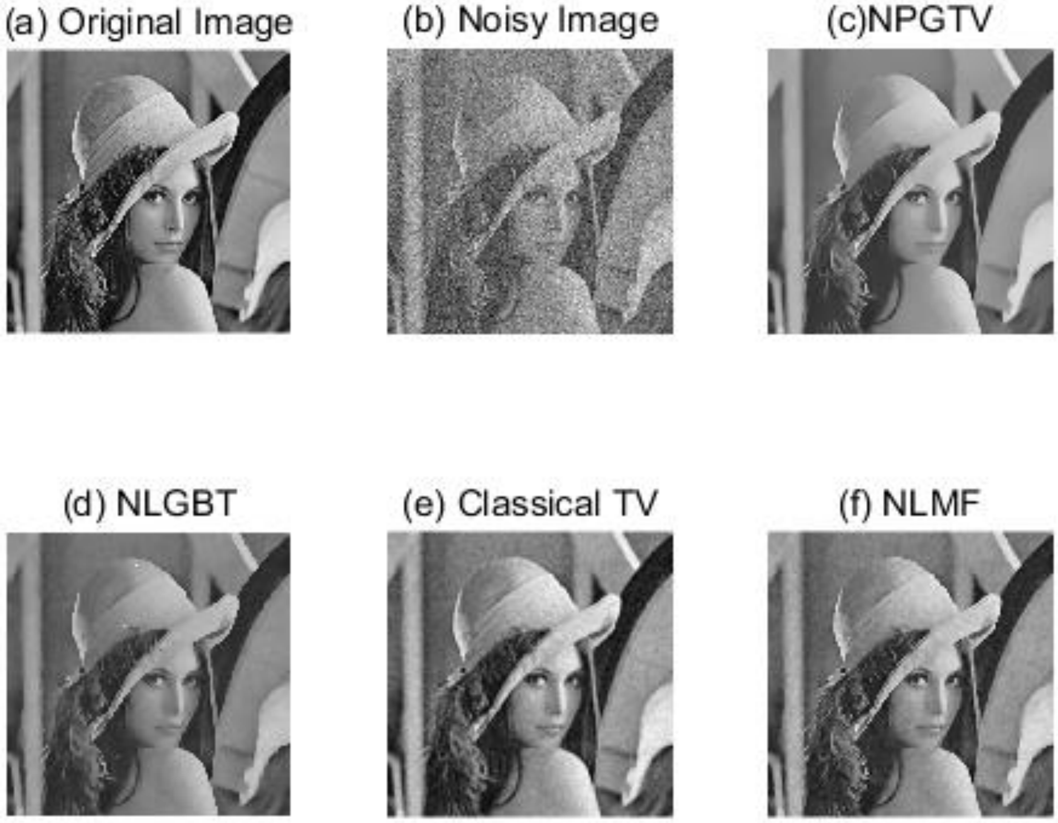

Fig. A. Results of different denoising methods on 512×512 Lena image. (a): Original image. (b): Noisy image corrupted by zero-mean Gaussian noise with 30db standard deviation. (c): NPGTV with k=5, λ=0.05, patch size 9×9. (d): NLGTV algorithm. (e): Classical TV. (f) NLMF with search window size 3×3, similar widow size 5×5 and the standard deviation is set to 0.1.

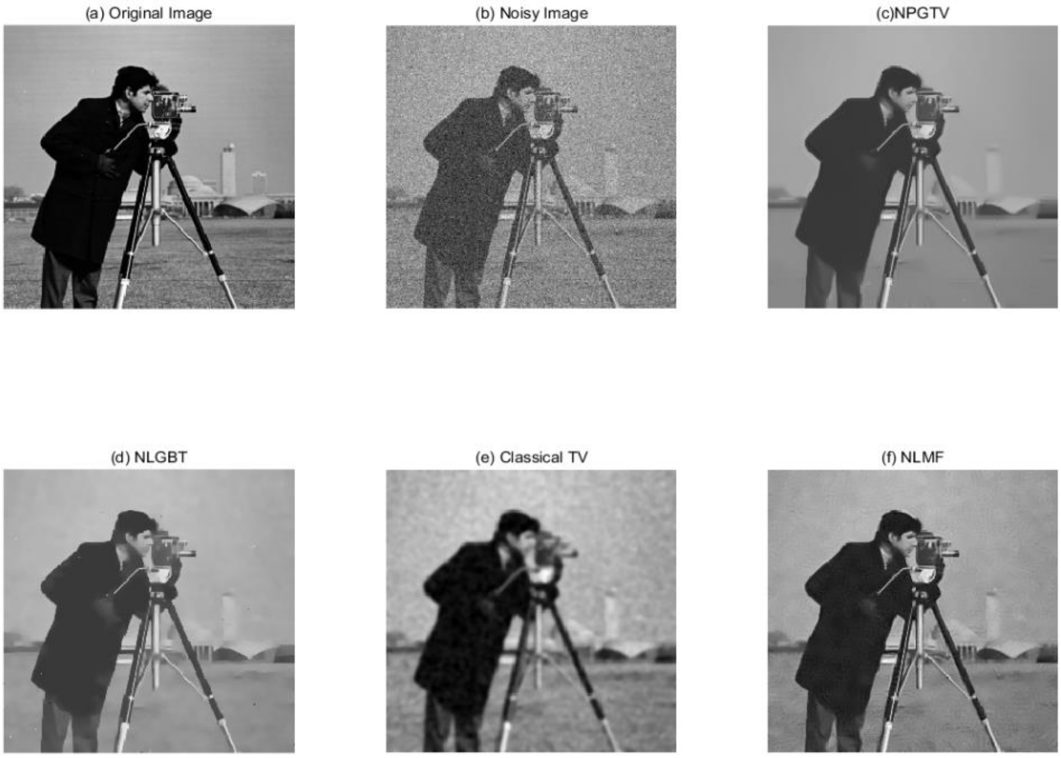

Fig. B. Results of different denoising methods on 256×256 **Lena image.** (a): Original image. (b): Noisy image corrupted by zero-mean Gaussian noise with 30db standard deviation. (c): NPGTV with k=5, λ=0.05, patch size 9×9. (d): NLGTV algorithm. (e): Classical TV. (f) NLMF with search window size 3×3, similar widow size 5×5 and the standard deviation is set to 0.1.

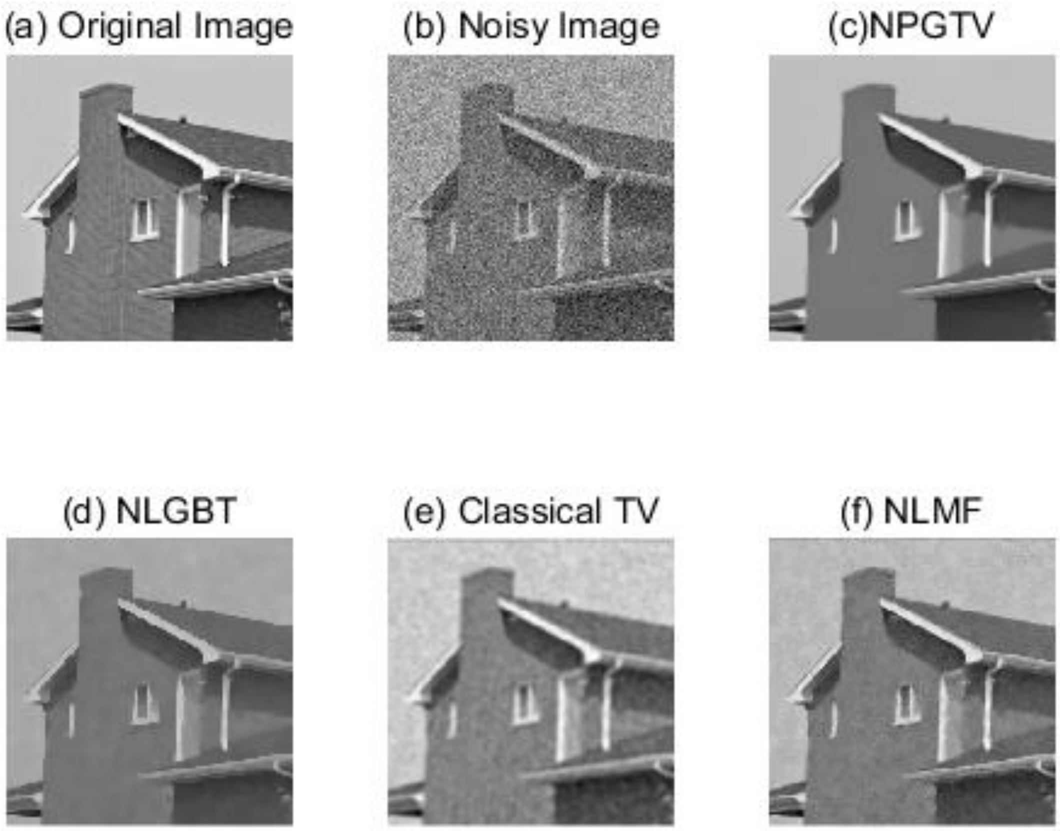

Fig. C. Results of different denoising methods on 256×256 House image. (a): Original image. (b): Noisy image corrupted by zero-mean Gaussian noise with 30db standard deviation. (c): NPGTV with k=5, λ=0.05, patch size 9×9. (d): NLGTV algorithm. (e): Classical TV. (f) NLMF with search window size 3×3, similar widow size 5×5 and the standard deviation is set to 0.1.

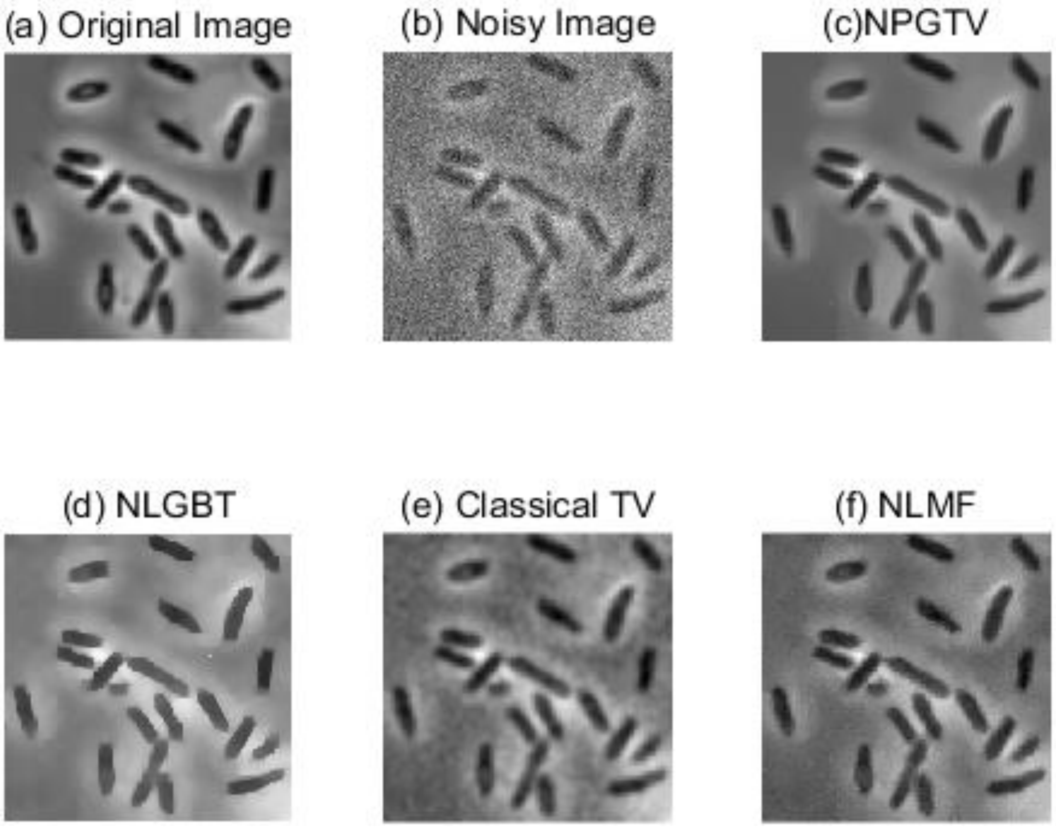

Fig. D. Results of different denoising methods on 176×176 Bacteria image. (a): Original image. (b): Noisy image corrupted by zero-mean Gaussian noise with 30db standard deviation. (c): NPGTV with k=5, λ=0.05, patch size 9×9. (d): NLGTV algorithm. (e): Classical TV. (f) NLMF with search window size 3×3, similar widow size 5×5 and the standard deviation is set to 0.1.

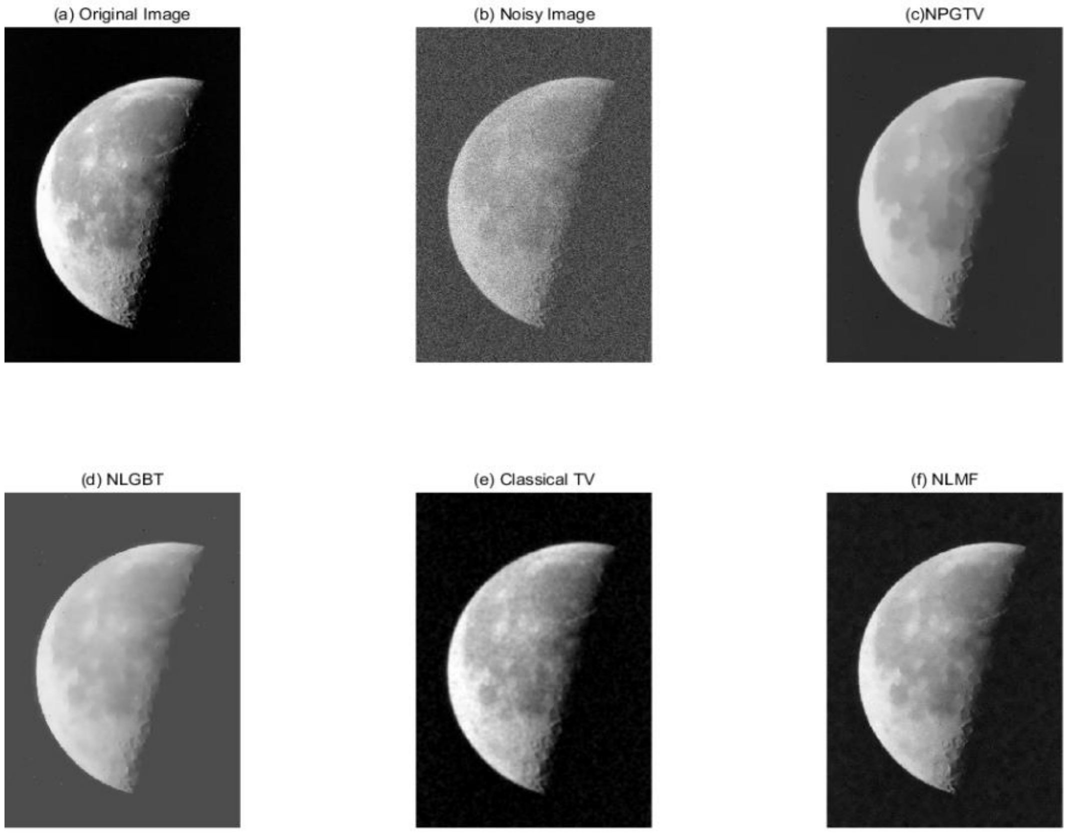

Fig. E. Results of different denoising methods on 537×358 Moon image. (a): Original image. (b): Noisy image corrupted by zero-mean Gaussian noise with 30db standard deviation. (c): NPGTV with k=5, λ=0.05, patch size 9×9. (d): NLGTV algorithm. (e): Classical TV. (f) NLMF with search window size 3×3, similar widow size 5×5 and the standard deviation is set to 0.1.
